# Deriving Heterospecific Self-Assembling Protein–Protein Interactions Using a Computational Interactome Screen

**DOI:** 10.1016/j.jmb.2015.11.022

**Published:** 2016-01-29

**Authors:** Richard O. Crooks, Daniel Baxter, Anna S. Panek, Anneke T. Lubben, Jody M. Mason

**Affiliations:** 1 Department of Biology and Biochemistry, University of Bath, Claverton Down, Bath BA2 7AY, United Kingdom; 2 Chemical Characterisation and Analysis Facility, University of Bath, Claverton Down, Bath BA2 7AY, United Kingdom

**Keywords:** PPI, protein–protein interaction, bCIPA, bZIP coiled-coil interaction prediction algorithm, SEC, size-exclusion chromatography, MS, mass spectrometry, protein–protein interactions, *de novo* design, heterospecific proteins, peptide, interactome screen

## Abstract

Interactions between naturally occurring proteins are highly specific, with protein-network imbalances associated with numerous diseases. For designed protein–protein interactions (PPIs), required specificity can be notoriously difficult to engineer. To accelerate this process, we have derived peptides that form heterospecific PPIs when combined. This is achieved using software that generates large virtual libraries of peptide sequences and searches within the resulting interactome for preferentially interacting peptides. To demonstrate feasibility, we have (i) generated 1536 peptide sequences based on the parallel dimeric coiled-coil motif and varied residues known to be important for stability and specificity, (ii) screened the 1,180,416 member interactome for predicted *T*_m_ values and (iii) used predicted *T*_m_ cutoff points to isolate eight peptides that form four heterospecific PPIs when combined. This required that all 32 hypothetical off-target interactions within the eight-peptide interactome be disfavoured and that the four desired interactions pair correctly. Lastly, we have verified the approach by characterising all 36 pairs within the interactome. In analysing the output, we hypothesised that several sequences are capable of adopting antiparallel orientations. We subsequently improved the software by removing sequences where doing so led to fully complementary electrostatic pairings. Our approach can be used to derive increasingly large and therefore complex sets of heterospecific PPIs with a wide range of potential downstream applications from disease modulation to the design of biomaterials and peptides in synthetic biology.

## Highlights

• Naturally occurring protein–protein interactions (PPIs) are highly specific.
• For designed PPIs, however, specificity can be notoriously difficult to engineer.
• We have computationally screened a vast interactome to derive four heterospecific PPIs.
• Eight peptides form four heterospecific coiled coils; all 32 off targets are disfavoured.
• The method can derive larger and increasingly complex sets of heterospecific PPIs

## Graphical abstract

**Figure:**
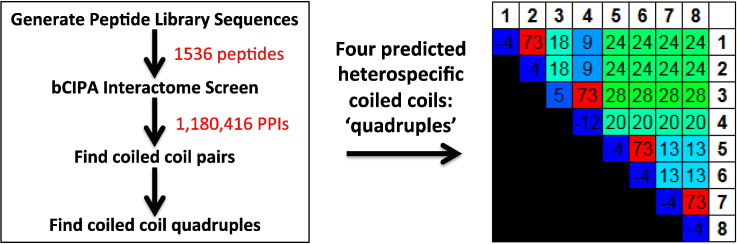

## Introduction

The number of hypothetical protein–protein interactions (PPIs) in a given protein interactome increases quadratically with the number of protein components, allowing for the creation of complex interaction patterns using only a relatively small number of proteins. The importance of PPI complexity has been recognised since the advent of genomics [1] but has recently gained considerable attention for its wide variety of potential applications in synthetic biology [2], [3]. For any given interactome, the affinity that one member has with another will vary, giving rise to specific interactions that can be formed between different peptide partners. We have focused our efforts on the development of software that can identify sets of peptides that are capable of forming heterospecific PPIs when combined. Resulting peptide sequences can then be tested in the laboratory to verify stability and specificity of interaction to demonstrate that the prediction software is effective. We anticipate that experimentally derived data can then be used to further improve and refine the accuracy of future versions of the prediction software.

Furthermore, predicting stability and specificity arising from protein folding and association from the primary sequence is a major goal in protein science that is becoming increasingly tractable for the parallel dimeric coiled-coil motif. However, whilst rules governing stability are reasonably well understood, those directing specificity are not [4], [5]. There is therefore considerable interest in PPI design strategies that consider both affinity of the interaction and multistate specificity. However, optimisation of PPIs is challenging since one needs to identify sequences that favour one state whilst leading to disfavoured states for a number of off-targets. Our approach starts with the bZIP coiled-coil interaction prediction algorithm (bCIPA) [6], [7], which unlike related qualitative algorithms [8], [9] is able to provide a quantitative measure of stability from the primary sequences of the peptides in the form of a predicted thermal melting (*T*_m_) value. bCIPA was derived using the natural interactome of the human Jun and Fos AP-1 family members. Coiled coils are a model system as they are encoded by a seven-residue repeat designated [abcdefg]_n_ that can be exploited to engineer stable and specific PPIs [10]. Subsequently, several groups have designed specific coiled-coil peptide components for rational design and engineering and synthetic biology. These have included design of antiparallel coiled coils [11], PPI inhibitors [12], [13], *de novo* designed pairs [3], [14], [15] and designed higher oligomeric states [16], [17]. In contrast to these studies, bCIPA functions to directly estimate the *T*_m_ between two parallel dimeric coiled-coil forming peptides based on sequence input data alone, thereby providing a quantitative estimate on interaction affinity. To do so, it uses scoring matrices, taking into account the energetic contribution to stability that is made by hydrophobic residues within the core [18], [19] and electrostatic residues [20], as well as the overall predicted helicity of the component peptides [21]. More recently, bCIPA has been used to assist in the design of short α-helical tectons for constructing multicomponent synthetic biological systems [22]. We have now expanded the capabilities of the bCIPA algorithm to create a high-throughput screening tool that can predict the *T*_m_ values for many millions of hypothetical coiled-coil pairs within an interactome simply by inputting the primary amino acid sequences. A related program then works with bCIPA by searching within the interactome to identify sets of coiled coils that are predicted to retain specificity for their cognate partners when all peptides within the set are combined. A major advantage of our approach is its ease of implementation relative to others. To demonstrate a proof of principle for the software, we have (i) created a virtual library of sequences containing semirandomised interfacial positions with residue options known to be important for driving stability and specificity of interaction, (ii) used this library to create an interactome of millions of hypothetical pairwise interactions in which all potential *T*_m_ values are calculated and (iii) searched within this interactome to identify a set of eight peptides that can form four heterospecific coiled-coil pairs (“quadruples”) when combined. This requires the software to compare all possible combinations to ensure that all of the 32 off-target interactions within the resulting eight-peptide interactome are disfavoured. This demonstrates the utility of the software in peptide design and other high-throughput applications, as well as allowing for future improvement of prediction accuracy as residue pairings and their contributions in sequence-specific settings become better described. We have tested the feasibility of the approach by generating a virtual library of 1536 peptides and screened the resulting 1,180,416 member interactome to derive four coiled coils that preferentially heterodimerise, generating high-affinity interactions that are specific in each other's presence. To validate this, we have synthesised one set of computationally identified sequences in the laboratory and undertaken biophysical characterisation experiments to verify that they perform as predicted by the software. The results for these and future experiments will be used iteratively to further develop and refine the accuracy of our software prediction. In doing so, we anticipate that four sets of heterospecific PPIs (quadruples—interactomes consisting of eight peptides) derived using this approach can be combined to create eight (octuplets—interactomes consisting of 16 peptides) and even greater numbers of heterospecific PPI sets. This in turn could provide a variety of versatile coiled coils for use in a wide range of applications, and we envisage that patterns arising from these studies can be used to devise rules that fast track the derivation of future heterospecific sequences.

## Results and Discussion

### Library design strategy

**Fig. 1:**
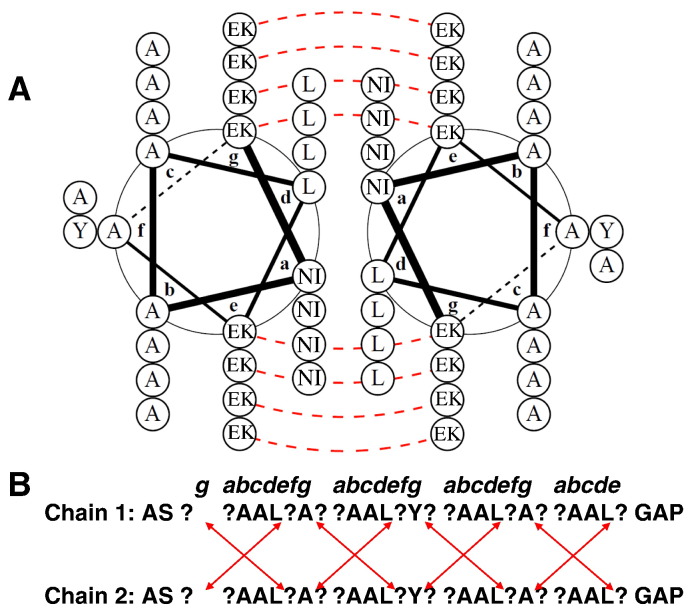
Helical wheel and linear peptide representations of the library design. Glu/Lys residue options were included at ***e*** and ***g*** positions and aimed at varying the level of electrostatic attraction at the dimeric interface. Ile/Asn options for ***a*** positions are also shown. Chains start with the ***g*** position and end with the ***e*** position to maximise potential electrostatic interactions. Position ***f***2 Y assists with concentration determination. Capping motifs have also been added. In total, 1536 sequences led to an interactome of 1,180,416 possible dimeric coiled coils. Helical wheel diagram generated using DrawCoil 1.0, http://www.grigoryanlab.org/drawcoil.

A 1536-member library of 32-residue peptides was designed based on sequence and library options that conform to a parallel dimeric coiled-coil motif [10]. The design principle of using a library of this length was to start with an N-terminal ***g*** position and finish with a C-terminal ***e*** position, maximising the electrostatic ***e***-***g*** contacts to eight, meaning that no noninteracting electrostatic residues arise at either terminus. Software was created to generate the library sequences (“Generate library”) using the description ***ph***AAL***p***A***ph***AAL***p***Y***p h***AAL***p***A***p h***AAL***p***, where each ***h*** signifies amino acid options of Asn/Ile at each core ***a*** position and each ***p*** signifies options of Glu/Lys at each electrostatic ***e*** and ***g*** positions (Fig. 1). Moreover, an option within the software was implemented to limit libraries to only those sequences that contained two Asn residues and two Ile residues. This reduced the possible number of core combinations from 16 to 6 (and therefore the number of peptides in the interactome screen reduced from 4096 to 1536). This offered the potential to optimise specificity by ensuring that either two or four of the core Asn/Ile residues become disfavoured (i.e., generate Asn-Ile pairings) in the undesired heterodimeric interactions [18], [19]. The software used to generate library sequences also allows the user to input a randomised sequence (either at the protein level or at the DNA level using degenerate codons) and specify which positions are to be scrambled and which amino acid options are to be offered at those positions. The program then lists every individual library member, which can be imported into downstream software applications.

Once all library sequences were listed, a second program (“Find pairs”) was used to search within the imported interactome to screen for two coiled coils (i.e., four peptides) that were predicted to be specific in each other's presence. Once these were identified, a third program (“Find quadruples”) was used to search for four coiled coils (i.e., eight peptides) that were predicted to be specific within each other's presence. The programs work to identify which interactions between a set of sequences do and do not take place and then assign a *T*_m_ and associated heatmap to the resulting interactome. They then allow the user to set the stringency for desired pairs according to their required stability, as well as the *T*_m_ cutoff for off-target interactions (homodimers and heterodimers). Given these sets of customised constraints, quadruples can be identified. In our case, the maximum acceptable predicted *T*_m_ for homodimers was set to 10 °C, the maximum *T*_m_ for desired heterodimers was set to 70 °C, the maximum *T*_m_ for undesired heterodimers was set as 20 °C and the minimum Δ*T*_m_ between desired and undesired pairs was set as 50 °C. This resulted in 72 separate groups of noninteracting pairs of coiled coils. The highest predicted *T*_m_ for the desired coiled coils was 73 °C and the highest predicted *T*_m_ for undesired coiled coils was 18 °C. The program additionally allows the user to specify other desired characteristics—in this case, that two Asn residues and two Ile residues should be found within every peptide. This was performed since, in desired coiled coils, Asn pairs are known to direct specificity (see the supporting information Including double Asn cores) [23], [24], [25]. Having identified two coiled coils of both high predicted affinity and specificity, the program can be further instructed to identify four coiled coils (or quadruples). In this case, minimum Δ*T*_m_ of 40 °C and a maximum undesired coiled coil *T*_m_ of 30 °C were specified within the software. This resulted in retaining 144 groups of noninteracting quadruples of coiled coils with the highest desired *T*_m_ of 73 °C and a lowest undesired coiled coil *T*_m_ of 28 °C.

### Analysis of the *de novo* derived sequences

**Fig. 2:**
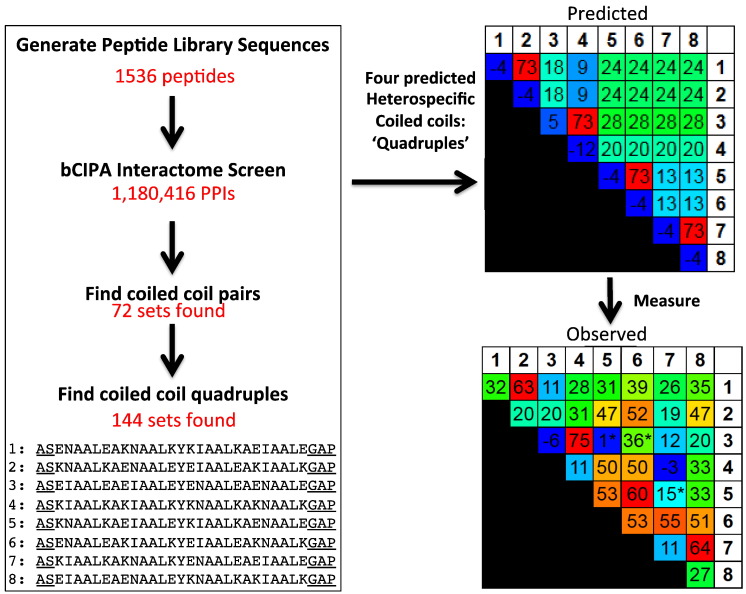
The development of a method to design sets of heterospecific coiled-coil interactions. Firstly, “Generate library” is used to create a complete list of the peptide library (in this case, a 4096-member peptide library was reduced to 1536). Next, the “bCIPA interactome screen” was used to predict the *T*_m_ of each potential coiled coil within the 1,180,416 pairwise PPI interactome. Next, “Find pairs” was used to identify sets of peptides that, according to the criteria input by the user, are predicted to be heterospecific when combined. In this case, 72 such sets were identified. Finally, “Find quadruples” works by combining pairs of coiled coils to identify groups of four coiled coils that are predicted to be heterospecific when combined. Here, 144 sets were identified. The eight-peptide set used in this study is shown, with additional capping sequences underlined. Shown on the right are the predicted and measured thermal melting values for all 36 possible pairs within the selected eight-peptide interactome. For a full description of the software, see the supporting information.

The above mentioned software resulted in 72 sets of coiled-coil pairs and 144 sets of coiled-coil quadruples. From the specific quadruples, we selected the set with the highest predicted desired *T*_m_ (73 °C; Fig. 2) and the largest predicted difference in *T*_m_ between desired and nondesired pairs (45 °C). The derived sequences were then synthesised as N- and C-capped peptides (additional residues underlined):

Peptide 1: ASENAALEAKNAALKYKIAALKAEIAALEGAP
Peptide 2: ASKNAALKAENAALEYEIAALEAKIAALKGAP
Peptide 3: ASEIAALEAEIAALEYENAALEAENAALEGAP
Peptide 4: ASKIAALKAKIAALKYKNAALKAKNAALKGAP
Peptide 5: ASKNAALKAEIAALEYKIAALKAENAALEGAP
Peptide 6: ASENAALEAKIAALKYEIAALEAKNAALKGAP
Peptide 7: ASKIAALKAKNAALKYENAALEAEIAALEGAP
Peptide 8: ASEIAALEAENAALEYKNAALKAKIAALKGAP

Inspection of all hypothetical helical wheels between peptides 1-to-4 and 5-to-8 (see Table 1 and Fig. S1) led to the following observations about the anticipated stabilities and specificities between the eight sequences. These observations were that (i) all desired heterospecific pairs (i.e., 1-2, 3-4, 5-6 and 7-8) contain two Asn-Asn and two Ile-Ile core pairings and all eight electrostatic interactions being favourable (i.e., all are Glu-Lys pairings), that (ii) all homodimeric off-target interactions contain the same two favoured Asn-Asn and two Ile-Ile core pairs but with all eight electrostatic interactions being unfavourable (i.e., all are Glu-Glu or Lys-Lys pairings) and that (iii) all intrapair heterodimeric off-target interactions (i.e., off-target interactions between peptides 1-to-4 and 5-to‐8) contain four Asn-Ile core pairs and four favourable and four unfavourable electrostatic interactions.

**Table 1:**
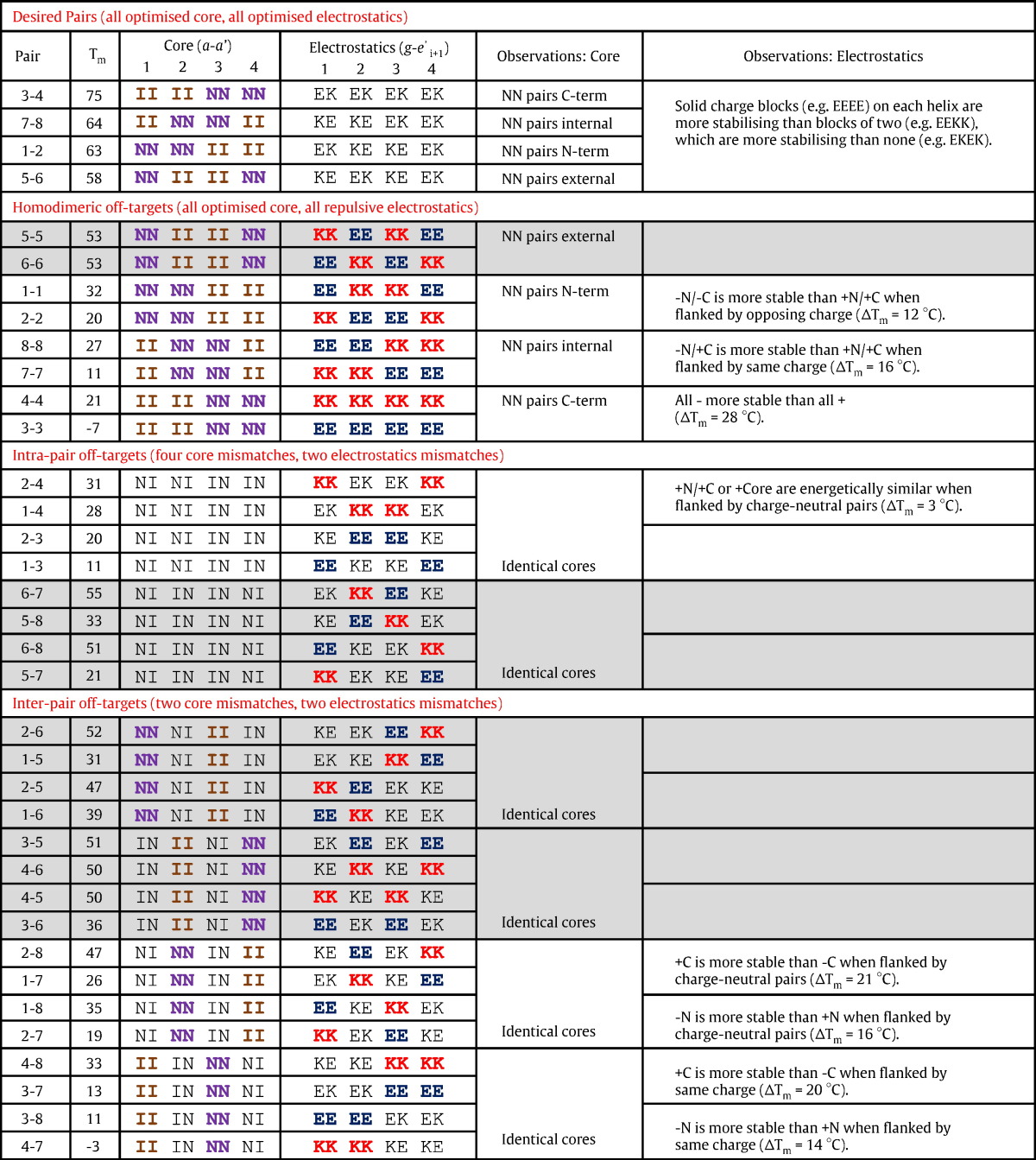
Core and electrostatic pairings for all 36 coiled-coil pairs.

All are grouped into desired pairs, intrapair off-target pairs and interpair off-targets pairs and ranked in order of *T*_m_ and according to core arrangement. Grey boxes show interactions including peptide 5 or 6 that may involve adoption of antiparallel conformations. Predicted energetic contributions to stability for heterospecific targets, homodimeric off-targets, heterodimeric off-targets (pairs) and heterodimeric off-targets (quadruples) are shown. Heterospecific pairs are predicted to have a Δ*G*_Core_ of − 23.2 kcal/mol based on free energy scored derived from a double mutant analysis [18], [19]. This is identical in homodimeric off-target pairs but is disfavoured for intrapair heterodimeric off-targets (ΔΔ*G* = + 21.2 kcal/mol) and interpair heterodimeric off-targets (ΔΔ*G* = + 10.6 kcal/mol). Similarly, heterospecific pairs are predicted to have a Δ*G*_Electrostatic_ of − 9.6 kcal/mol based on free energy scores [20]. This is strongly disfavoured in homodimeric off-target pairs (ΔΔ*G* = + 7.2 to + 12.8 kcal/mol) and is disfavoured, although less so, for intrapair and interpair heterodimeric off-targets (ΔΔ*G* = + 3.6 to + 6.4 kcal/mol). Inspection of these energies shows that (i) heterospecific pairs contain two II and two NN core pairings and fully complementary electrostatic ***e***-***g*** pairings, that (ii) homodimer instability is driven entirely by electrostatic repulsion, that (iii) off-targets within interactomes of peptide 1-4 or 5-8 are disfavoured driven mostly by the core residues (at a ratio of approximately 3–6:1) and that (iv) off-target interactions between peptides 1-4 and 5-8 are disfavoured driven mostly by the core residues (at a ratio of approximately 1.5–3:1). Sequence-specific E/K patterns are discussed with reference to normalised core configurations.

[Core free energy scores II = − 9.2 kcal/mol, NN = − 2.4 kcal/mol (Δ + 6.8) and NI = − 0.5 kcal/mol (Δ + 8.7)] [18], [19].

[Electrostatic free energy scores EK/KE = − 1.2 kcal/mol, EE = + 0.4 kcal/mol (Δ + 1.6) and KK = − 0.3 kcal/mol (Δ + 0.9)] [20].

When combining the anticipated interpair interactions between peptide sets 1-4 and 5-8 (and all of which are therefore off-target), the general observations were that (i) all pairs contain one Asn-Asn, one Ile-Ile and two Asn-Ile core pairs and four favourable/four unfavourable electrostatic interactions, as was the case for the intrapair off-target heterodimeric interactions. In addition, the two favourable/two unfavourable electrostatic interactions were found to cycle through every possible pattern (see Table 1) in these off-target pairs.

### Circular dichroism spectra and thermal denaturation experiments

**Fig. 3:**
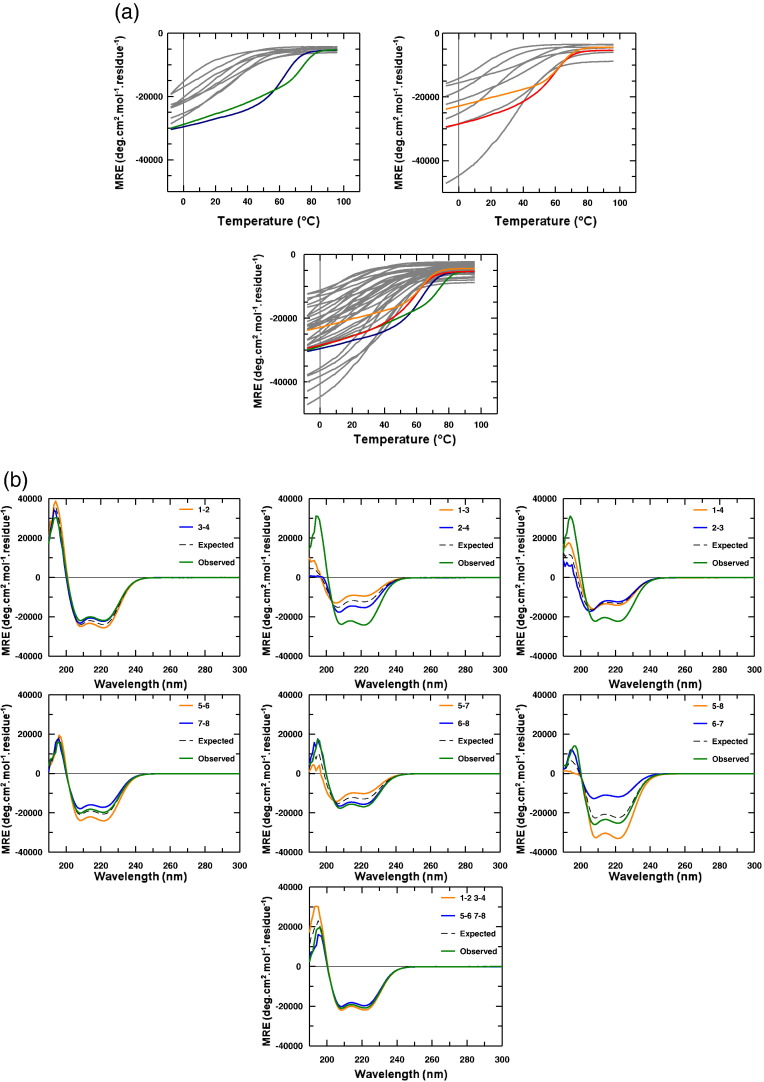
Shown are (a) thermal stability of peptide pairs measured by using temperature dependence of the CD signal at 222 nm and (b) dimer exchange experiments. For thermal denaturation, all 36 peptide pairs are shown, with heterospecific pairs colour coded (1-2, blue; 3-4, green; 5-6, red; 7-8, orange). For dimer exchange experiments, different peptide combinations were mixed at 20 °C. Mixing 1-2 (orange; *T*_m_ 63 °C) with 3-4 (blue; *T*_m_ 75 °C) resulted in no exchange, with the superimposition of the calculated summed data (black hash) with the observed helix mixture (green). In contrast, mixing 1-3 with 2-4 or 1-4 with 2-3 resulted in major increases in helicity compared to the averaged spectra. The pattern was similar (although less pronounced) for peptide 5-to-8. Combining an equimolar mixture of 1-2-3-4 with 5-6-7-8 resulted in no further gain over the average of the individual signals, indicating that components of peptide mixture 1-to-4 do not switch to interact with peptide mixture 5-to-8 and that, accordingly, the four coiled-coil pairs are specific within the entire interactome.

To demonstrate that the *in silico* generated sequences are specific in the laboratory, we synthesised and characterised all eight peptides. The circular dichroism (CD) spectra were used to confirm that all samples were α-helical. Thermal denaturation experiments were then used to establish the *T*_m_ value for each coiled coil within the eight-peptide interactome and hence the relationship between predicted and measured values (Fig. 3a). The four pairs predicted to be heterospecific were verified experimentally, with predicted *T*_m_ values of 73 °C found to be accurate to within 13 °C (Table 1). Thermal melting data for peptides within set 1-4 demonstrated the off-target interactions to be no greater than 32 °C, providing Δ*T*_m_ values (desired—most stable off-target) of 31 and 43 °C compared to 55 °C predicted. The 32 off-target interactions (14 off-targets for each desired interaction) although more variable, with predicted *T*_m_ values of − 3 °C to 55 °C, importantly ensured that the 1-2, 3-4, 5-6 and 7-8 were heterospecific as designed.

### Experimental characterisation of peptide pairs

To demonstrate that the four desired pairs (i.e., 1-2, 3-4, 5-6 and 7-8) were heterospecific when mixed, we undertook a series of dimer exchange experiments. In these experiments, seven CD spectra were collected using both the predicted and measured interactome data as a guide (Fig. 2, Fig. 3a). In the first experiment, scans for peptide mixtures 1 + 2 (orange) and 3 + 4 (blue) were taken individually before being mixed (i.e., 1 + 2 + 3 + 4; green). The same experiment was repeated starting with 1 + 3 and 2 + 4 peptide mixture scans followed by 1 + 4 and 2 + 3 peptide mixture scans. These were undertaken to demonstrate that only in the second and third instances is a large increase in helical signal observed as individual helices exchange to form the most stable pairs, that is, to give the two desired heterospecific coiled-coil pairs that are already present in the first experiment. Similarly, a 5-6 + 7-8 scan, a 5-7 + 6-8 scan and a 5-8 + 6-7 scan demonstrates that only in the second and third instances was an increase in helicity above the calculated average observed, demonstrating that 5-6 and 7-8 are specific dimers within this set. Finally, combining an equimolar mixture of peptides 1-to-4 with peptides 5-to-8, resulted in no further gain over the average of these individual signals, indicating that components of peptide mixture 5-to-8 do not switch to interact with components of peptide mixture 1-to-4 and that accordingly the four coiled coils are specific within the entire interactome.

**Fig. 4:**
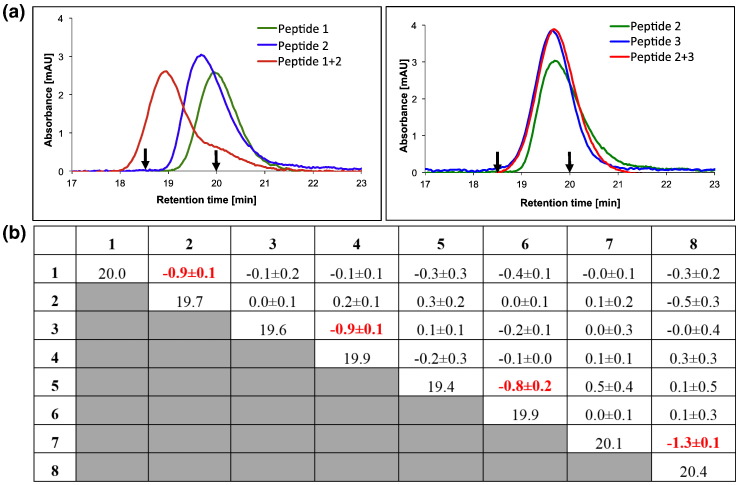
SEC experiments. Shown are (a) SEC profiles for interacting and noninteracting peptides. Left: A peak at approximately 19 min for a 1-2 mixture represents a dimeric sample whilst component peptides 1 and 2 generate a peak at approximately 20 min, indicating monomeric samples. Right: Peptides 1 and 3 and mixture 1-3 all elute at approximately 20 min, indicating that all of these samples exist in monomeric form. Arrows show previously characterised controls with elution times for a 32mer Fos monomeric peptide (20 min) and a 37mer cJun-FosW heterodimer (18.5 min). (b) Summary of all elution times for homomeric (all monomeric) samples and the net change to these times upon mixing. As can be seen, for the four desired pairs (1-2, 3-4, 5-6 or 7-8), this results in elution of the sample 1 min earlier than for the eight individual component peptides or indeed for the other 24 off-target mixed samples and is consistent with the formation of a dimer. These experiments provide strong evidence for heterospecificity of the four designed coiled coils.

As a further demonstration of correct peptide pairing, we used size-exclusion chromatography (SEC). Monomeric elution profiles were superimposable and occurred at approximately 20 min. These were in contrast to the dimeric profiles, which eluted at approximately 19 min. In both cases, the elution profiles were consistent with that predicted monomer/dimer patterns (Fig. 4). As controls, we used two peptides of similar length that have been previously characterised and shown to exist in either monomer form or as a parallel dimeric coiled coil [6]. Lastly, peptide 1-8 in isolation and peptides 1-2, 3-4, 5-6 and 7-8 mixtures were analysed using ultrahigh-resolution time-of-flight mass spectrometry (MS). Consistent with SEC, MS experiments demonstrated that all of the peptides in isolation exist in monomeric (3.3 kDa) rather than homodimeric form and that the desired heterodimeric pairs (6.6 kDa) form only when the four appropriate peptides are combined.

### Successes in the design

With the use of low-complexity options where energetics of pairing are relatively well understood [18], [19], [20], it is possible to generate more complex sequence-specific combinations of hydrophobic and electrostatic patterns to confer specificity. The additional benefit of generating insight into the role of these contributions in different sequence settings is that it will allow for further refinement of our software and consequently of the derived sequences in future design iterations. The tools were created to search for the required sequence complexity that dictates the formation of highly specific heterodimers that deviate from the simple acid–base technique used in a “Peptide Velcro” approach [26]. In addition, the approach is distinct from those taken by the Keating group [12], [13], which uses more complex computational algorithms using integer linear programming and cluster expansion to generate peptide ligands for defined targets/off-targets. In contrast, our software is very simple to use and differs in that it searches within large user-defined peptide sets to identify and provide quantitative outputs in the form of a *T*_m_ to derive sets of eight peptides predicted to form heterospecific coiled coils. In the example used, each of the four desired coiled coils had predicted *T*_m_ values of 73 °C, with the highest of the 14 potential off-target interactions for any given coiled-coil pair predicted to be 18 °C (intrapair off-targets) and 28 °C for coiled-coil quadruples (interpair off-targets). This increase in off-target *T*_m_ within this library was due to the difficulty in maintaining specificity when the number of off-target interactions increased from 8 to 32 hypothetical pairs.

Analysis of the core and electrostatics demonstrated that, as expected, the most favoured contributions are found in the desired pairs, where the core is optimised to contain two Ile-Ile and two Asn-Asn pairs (predicted Δ*G* = − 23 kcal/mol; see Table 1). All eight electrostatic interactions are favourable (predicted Δ*G* = − 9.6 kcal/mol; see Table 1). All eight homodimers are predicted to be disfavoured, with this effect driven by the loss of all eight electrostatic interactions (predicted ΔΔ*G* = + 7.2 to + 12.8 kcal/mol) and with the core interactions unchanged relative to the heterospecific pairs. All heterodimeric off-targets within pairs (i.e., within set 1-4 or 5-8) are disfavoured by both the core (four disfavoured Asn-Ile pairs; predicted ΔΔ*G* = + 21.2 kcal/mol) and to a lesser extent the electrostatic contribution, which contains four favourable and four unfavourable interactions (predicted ΔΔ*G* = + 3.6 to + 6.4 kcal/mol). All heterodimeric off-targets within the interpair combinations are also disfavoured to the same extent by the electrostatic contributions (predicted ΔΔ*G* = + 3.6 to + 6.4 kcal/mol) and by a weaker destabilisation of the core interactions (with one Asn-Asn, one Ile-Ile and only two disfavoured Asn-Ile pairs; predicted ΔΔ*G* = + 10.6 kcal/mol) relative to intrapair combinations. Thermal melt and dimer exchange studies demonstrate that four heterospecific pairs can be derived upon incubation of the eight-peptide sequences. This finding is further supported by SEC studies on all 36 hypothetical pairs within the interactome and demonstrates that only the four desired pairs dimerise (confirmed by MS) and that peptides within all 32 off-targets exist in the monomeric form. Heterospecificity is driven by electrostatic repulsions for homodimers (with the core pairings identical with the desired pairs) and predominantly by destabilisation of core residue interactions for intrapair off-targets (by a core:electrostatic ratio of about 6:1) and similarly, albeit less, for interpair off-targets (by a ratio of around approximately 1.5–3:1). This means that *T*_m_ values are higher and the stringency of specificity is slightly reduced for interpair off-targets.

### Caveats in the design

We identified several instances within the experimentally measured interactome where the *T*_m_ was underestimated. In particular, these relate to homodimers and heterodimers involving peptides 5 and 6. Further inspection of these sequences suggested that dimers may be stabilised by their ability to adopt antiparallel species. For example, homodimers of peptide 5-8 in the antiparallel orientation all have fully favourable electrostatic complements. During software development and consequent identification of peptide pairs and quadruples, antiparallel dimers were not predicted to form. This was because Asn-Asn core pairings between ***a-a′*** residues that make the major energetic contribution to coiled-coil specificity in the parallel orientation are unable to do so in the antiparallel orientation. Rather, buried polar interactions in antiparallel dimers take place between ***a-d′*** residues and would therefore not be considered possible in this system [27], [28]. Thus, despite the favourable ***e-e′*** and ***g-g′*** pairings in the antiparallel orientation, these structures were not be predicted to be of high stability. It is interesting that whilst 5 and 6 form stable homodimers (*T*_m_ = 53 °C), 7 and 8 do not (*T*_m_ = 11–27 °C). The main difference being that in 5-5 and 6-6 pairs the four core Asn residues are located at the helix termini, where solvent exposure is greater, leaving four Ile buried next to Leu residues in the centre of the helices. In contrast, 7-7 and 8-8 pairs show a reversal of this pattern, resulting in the four Asn residues buried in the centre of the helices and in proximity to the same Leu residues, leading to much less stability. A consequence of this is that off-target interactions for peptide 5-8 were found to be higher than anticipated, at up to 55 °C, providing Δ*T*_m_ values (desired—most stable off-target) of only 5 and 9 °C compared to the predicted value of 60 °C. This increase is likely to be due to the unpredicted stability for homodimers or heterodimers with peptide 5 or 6. Reassuringly however, thermal melt analysis demonstrated the 5-6 heterodimer to be more stable than its respective homodimers or heterodimers containing these peptides, indicating that this complex forms preferentially. This was further confirmed by SEC analysis, which demonstrated dimerisation only in the case of the 5-6 pair. Similarly, for the eight-peptide interactome between peptides 1-to-4 and 5-to-8, all four peptide pairs were found to be heterospecific as predicted by the “Find pairs” and “Find quadruples” software, with all 32 off-targets mixtures existing as monomers. With regard to the accuracy of our predictions, we find a very good correlation for the interactome between peptides 1-to-4 (*r*^2^ = 0.70; see the supporting information). Owing to the antiparallel issues discussed above, the correlation coefficient is poor for the interactome between peptides 5-to-8 (*r*^2^ = 0.29) and consequently for the complete interactome between peptides 1-to-8 (*r*^2^ = 0.26).

### Sequence-specific findings

Throughout the study, we observed variations in affinity from energetically equivalent core and electrostatic pairings (Table 1), leading us to search for preferred sequence-specific combinations that could be utilised in the future to maximise desired pair stabilities whilst destabilising the nondesired off-targets. This has led to the following observations: ***Desired pairs*** displayed measured *T*_m_ values that varied from 58 to 75 °C, suggesting that, for fully complementary electrostatic pairings, solid blocks of all acidic (EEEE) or all basic heptads (KKKK) on ***e/g*** residues in one helix are favoured over blocks of two acidic and two basic heptads (EEKK or KKEE), which in turn are favoured over alternating charge heptads (EKEK or KEKE; see Table 1, Desired pairs). ***Homodimeric off-targets*** shared the same energetically favourable core as the desired pairs but with fully repulsive electrostatic pairings. When comparing identical core configurations, we observed that, in the cases of two acidic and two basic heptads, an acidic N/C terminus was more stable than a basic N/C terminus (EKKE > KEEK 1-1–2-2, Δ*T*_m_ = 12 °C). Similarly, an acidic N-terminus and basic C-terminus was more stable than *vice versa* (EEKK > KKEE 8-8–7-7, Δ*T*_m_ = 16 °C). Finally, All acidic residues were more stable than all basic (EEEE > KKKK 4-4–3-3, Δ*T*_m_ = 28 °C). ***Heterodimeric off-targets*** contained two attractive and two repulsive electrostatic pairs, meaning that repulsive pairs flanked either other repulsive pairs or charge-neutral pairs. When normalised against identical core configurations, a basic N/C-terminus was found to be energetically similar to internal residues (KxxK ≈ xKKx; 2-4–1-4, Δ*T*_m_ = 3 °C). Internal acidic residues were found to be slightly less destabilising than an acidic N/C-terminus (xEEx > ExxE 2-3–1-3, Δ*T*_m_ = 9 °C). When the N-terminus was charge neutral, basic residues were found to be more stabilising than acidic at the C-terminus, irrespective of whether they flanked charged or charge-neutral pairs (xExK > xKxE 2-8–1-7, Δ*T*_m_ = 21 °C; xxKK > xxEE, Δ*T*_m_ = 20 °C). Similarly, when the C-terminus was charge neutral, acidic residues were found to be more stabilising than basic at the N-terminus, irrespective of whether they flanked charged or charge-neutral pairs (ExKx > KxEx 1-8–2-7, Δ*T*_m_ = 16 °C; EExx > KKxx, Δ*T*_m_ = 14 °C). The different observations relative to homomeric off-target electrostatic sequence energies may owe to the fact that charge repulsions are flanked by charge-neutral pairs.

In general terms therefore, when compared against identical core configurations, it was more destabilising when basic residues were placed at the N-terminus, with the same effect (although less pronounced) when acidic charges were placed at the C-terminus. This is because the presence of either will destabilise the helix by failing to counter the helix macrodipole and prevent terminal fraying of the peptides. This has been observed in helices within globular proteins [29] where Glu was found to be favoured at positions N2-N3, and Lys was found to be favoured at positions C1-C3. It has also been shown in short designed single α-helices where Glu at position N2 stabilised helix formation, whilst Lys destabilised it [30], with the opposite effect apparent at the C-terminus [31]. Therefore, desired pairs can be maximally stabilised by introducing blocks of two same-charge heptads. Similarly, off-targets can be maximally destabilised by avoiding acidic N-terminal heptads (or promoting basic N-terminal heptads) and avoiding basic C-terminal heptads (or promoting acidic C-terminal heptads). Incorporating the above mentioned sequence-specific observations into future designs may assist in providing additional energy gaps between the desired and off-target states by selecting only the most stable desired sequences and only the least stable off-target sequences.

### Future direction

To avoid the possibility of antiparallel alignments identified by our “Find pairs” and “Find quadruples” software, we have now added an additional feature to the interactome screen. Owing to the exclusivity of Leu at ***d*** positions, the core is already optimised for parallel alignment. Instead, we have enabled a feature that searches for and removes homodimers that generate full electrostatic complementarity in the antiparallel orientation. This removes additional stability for these otherwise permissible antiparallel pairs. It also reduces the search time of the algorithm by increasing the stringency in the selection of the initial sequences that are processed into interactions and consequently reduces the size of the search required to find pairs and quadruples. Owing to how the library has been made smaller, removing sequences at intelligent steps to maximise computational efficiency, the method is very rapid, being able to identify the quadruples we have described from the initial 1536 sequences in under 2 min, thus showing that the method can be utilised for much larger libraries within a sensible timescale.

We envisage that rules generated by the software can be applied as prerequisites to fast track the derivation of future heterospecific sequences. Characterising these sequences will in turn help us to test the accuracy of predictive models and, consequently, our understanding of this class of PPI motif. As a proof of principle of the system, we have derived four heterospecific coiled coils using 32mer sequences. No less important is that the authors now have a hypothesis for how to generate more complex systems and believe that the software can be used to derive six, eight or even higher numbers of heterospecific pairs with peptides of varying length. This could be achieved for instance by searching within the multiple groups of noninteracting quadruples of coiled coils identified by the software to find greater numbers of heterospecific dimers. In the system tested, four ***a-a′*** pairs and eight ***e-g*** pairs were considered sufficient to generate specificity for four coiled-coil pairs with a predicted *T*_m_ of 45 °C. To permit higher numbers of specific coiled coils with comparable stringency, it is likely that longer peptide lengths and increased knowledge of coiled-coil structure stability from emerging databases and evolutionary profiles [32] will be needed to generate the higher numbers and more diverse offerings of ***a-a′*** and ***e-g*** pairs that can confer the required specificity. Creation of such heterospecific PPIs using this approach has the potential to vastly expand the synthetic biologist's toolkit in creating modular component peptide parts that can be used to create specific peptide tags [33] and biomaterials such as novel functional self-assembling structures for application in medical and material sciences [34]. This includes using coiled-coil segments as building blocks to allow the self-assembly of larger structures such as fibres [35], [36] or hydrogels [37]. Such structures could also be used to create novel signalling pathways [38] or for the creation of novel protein folds not observed naturally that can be harnessed by arranging side chains to create artificial catalytic sites [39]. We believe that our findings contribute to the deeper understanding of PPI specificity needed to address these areas with the required accuracy.

## Methods

### Design rationale

Peptides were semirandomised at ***a***, ***e*** and ***g*** positions within the heptad repeat (Fig. 1). Options of Glu and Lys were included at all ***e*** and ***g*** positions. Lys was used since it has comparable performance to Arg in terms of helicity and forming electrostatic interactions, but is easier to incorporate into synthetic peptides. Gln, which has been used in previous libraries and designs [6], [40], was omitted since it interacts favourably with both acidic and basic residues and is not therefore expected to confer significant specificity to pairings and would be expected to be selected out during the screening. At ***d*** positions, Leu was maintained throughout as these are known to assist in driving the formation of parallel and dimeric coiled-coil species. At ***a*** positions, the residues were semirandomised to Asn and Ile. These residues provide the greatest specificity distinction between core position residues based on double mutant analyses [19], with both Asn-Asn (− 2.4 kcal/mol) and Ile-Ile pairs (− 9.2 kcal/mol) significantly more favourable than an Asn-Ile pair (− 0.5 kcal/mol). These energetic values are anticipated to give a specificity enhancement caused by favourable alignment relative to misaligned residues. Therefore, Asn-Asn pairing confers specificity because the hydrogen bonding benefit outweighs the lack of stability and limits oligomeric states to dimers [24], [41]. Asn-Asn and also Ile-Ile ***a-a′*** pairs are predicted to stabilise the derived peptides as dimers rather than higher-order oligomers or antiparallel coiled coils, where Asn-Asn core pairings are also not found [42]. This is because ***a-a′*** and ***d-d′*** contacts occur in parallel but not antiparallel coiled coils, meaning that an interaction between equivalent Asn residues in a homodimer will favour a parallel alignment [43]. Furthermore, it is anticipated that alignment of Asn residues in core positions will stabilise a particular axial alignment and prevent alternative axial alignments causing unexpected interaction patterns.

### *In silico* library screening

The *in silico* library was created using *Generate Library Sequences* (see the supporting information) to list each user-defined member of the library in a sequential manner. This library was next screened using the *bCIPA interactome screen* engine (see the supporting information), which has been developed to screen interactomes of sequences using bCIPA [6], [7] and derive a heatmap for millions of hypothetical peptide pairs. A 4096 peptide interactome was reduced to 1536 by specifying that a minimum of two Asn and two Ile residues are required at ***a*** positions to assist in imposing specificity. The resulting 1,180,416 hypothetical pairwise interactions within it were next screened using *Find pairs* (see the supporting information) to identify groups of four sequences that when placed together would be predicted to form heterospecific dimeric interactions, known as “pairs”. These pairs could then be further screened within the same page (using *Find quadruples*) to identify groups of eight sequences which when placed together in solution would again be predicted to form four heterospecific dimeric interactions, known as “quadruples”.

### Sequence screening protocol

Sequences that met the conditions of the initial constraints described (specificity against homodimerisation and the requirement of containing two Asn residues) were retained for the interactome screen. Elimination of sequences that do not fulfil these requirements at the outset reduces computational load, allowing even larger libraries to be screened than in the presented example. Each new sequence that satisfied these criteria and was added to the array was screened using the *bCIPA interactome screen* engine for interaction affinity with every other sequence in the array at the time the sequence was added. This prevents any repeated calculations so that each interaction is only calculated once (i.e., not bidirectionally). The results of these calculations were stored in the database, but only if the affinity of those interactions exceeded the minimum specified desired affinity of the desired heterospecific pairs (in this case, set to 70 °C). Thus, this database was a list of pairs of sequences that could potentially form heterospecific pairings. Interactions in this database, with a *T*_m_ greater than the minimum allowed in the input, were paired with each other iteratively, with a computational load-saving requirement that excluded pairs from being screened against one another where those pairs contained any of the same peptides (e.g., an interaction between peptides 1 and 2 could not be paired with an interaction between peptides 1 and 3 since peptide 1 appears in both interactions such that the pairs would not be specific, as there is clear cross-talk without needing to quantify the interactions). Potential pairs that did not have any identical sequences were paired iteratively, in a similar manner to identifying the peptide pairs. However, instead of a simple bCIPA calculation, a mini-interactome was created for each potential pair and the *T*_m_ calculations of interactions contained therein were checked against a user-specified maximum undesired *T*_m_. Any undesired interactions with a predicted *T*_m_ of greater than 20 °C meant that the group of sequences was rejected as a specific pair. Where sequences met these criteria, they were retained as a pair of noninteracting coiled coils identified in the interactome. “Quadruples” were next identified by comparing sets of pairs to one another in a similar manner as previously (by cross-checking identified noninteracting pairs). However, in the case of quadruples, the increased stringency meant that a higher maximum *T*_m_ for an undesired interaction was used, in this case, 30 °C, with a minimum Δ*T*_m_ (desired − nondesired) of 40 °C.

### Homodimer removal

In order to preserve system resources and to limit the interactome screen to within useful search space, we removed sequences that were not expected to produce specific coiled coils. Search constraints for the interactome excluded all sequences that were predicted to have a homodimeric *T*_m_ greater than 10 °C at the earliest opportunity (as sequences are imported into the script). Sequences retained at this stage were stored in a MySQL database, together with the Williams helicity score [21] (to save recalculation).

### Peptide synthesis

Rink amide ChemMatrix™ resin was obtained from PCAS Biomatrix, Inc. (St.-Jean-sur-Richelieu, Canada); Fmoc l-amino acids and 2-(1*H*-benzotriazole-1-yl)-1,1,3,3-tetramethyluronium hexafluorophosphate or benzotriazol-1-yl-oxytripyrrolidinophosphonium hexafluorophosphate were obtained from AGTC Bioproducts (Hessle, UK); all other reagents were of peptide synthesis grade and obtained from Thermo Fisher Scientific (Loughborough, UK). Peptides were synthesised on a 0.1-mmol scale on a PCAS ChemMatrix™ Rink amide resin using a Liberty Blue™ microwave peptide synthesiser (CEM; Matthews, NC) employing Fmoc solid-phase techniques (for review, see Ref. [44]) with repeated steps of coupling, deprotection and washing (4 × 5 ml dimethylformamide). Coupling was performed as follows: Fmoc amino acid (5 eq), 2-(1*H*-benzotriazole-1-yl)-1,1,3,3-tetramethyluronium hexafluorophosphate or benzotriazol-1-yl-oxytripyrrolidinophosphonium hexafluorophosphate (4.5 eq) and diisopropylethylamine (10 eq) in dimethylformamide (5 ml) for 5 min with 35-W microwave irradiation at 90 °C. Deprotection was performed as follows: 20% piperidine in dimethylformamide for 5 min with 30-W microwave irradiation at 80 °C. Following synthesis, we acetylated the peptide—acetic anhydride (3 eq) and diisopropylethylamine (4.5 eq) in dimethylformamide (2.63 ml) for 20 min—and then cleaved it from the resin with concomitant removal of side-chain-protecting groups by treatment with a cleavage mixture (10 ml) consisting of TFA (95%), triisopropylsilane (2.5%) and H_2_O (2.5%) for 4 h at room temperature. Suspended resin was removed by filtration, and the peptide was precipitated using three rounds of crashing in ice-cold diethyl ether, vortexing and centrifuging. The pellet was then dissolved in 1:1 MeCN/H_2_O and freeze-dried. Purification was performed by RP-HPLC using a Phenomenex Jupiter Proteo (C18) reverse-phase column (4 μm, 90 Å, 10 mm inner diameter × 250 mm long). Eluents used were as follows: 0.1% TFA in H_2_O (a) and 0.1% TFA in MeCN (b). The peptide was eluted by applying a linear gradient (at 3 ml/min) of 5–70% B over 40 min. Fractions collected were examined by electrospray MS, and those found to contain exclusively the desired product were pooled and lyophilised. Analysis of the purified final product by RP-HPLC indicated a purity of > 95%.

### Circular dichroism

CD was carried out using an Applied Photophysics Chirascan CD apparatus (Leatherhead, UK) using a 200-μl sample in a CD cell with a 1-mm path length. Samples contained 150 μM total peptide (Pt) concentration at equimolar concentration for heterodimeric solutions (i.e., 75 μM per peptide) and suspended in 10 mM potassium phosphate and 100 mM potassium fluoride at pH 7 1 h prior to analysis. The CD spectra of samples were scanned between 300 nm and 190 nm in 1-nm steps, averaging 0.5 s at each wavelength. Three scans at 20 °C were averaged to assess helical levels and coiled-coil structure.

### Thermal denaturation experiments

Thermal denaturations were performed at 150 μM Pt in 10 mM potassium phosphate and 100 mM potassium fluoride, pH 7, using an Applied Photophysics Chirascan CD instrument (Leatherhead, UK). The temperature ramp was set to stepping mode using 1 °C increments and paused for 30 s at each temperature before measuring ellipticity at 222 nm. For all temperature denaturation experiments, data collection was started at − 8 °C, and at this temperature, the peptide solutions remained aqueous. Data collection continued to 95 °C. Data points for thermal denaturation profiles represent the averaged signal after 4 s of data collection. Melting profiles (see Fig. 3a) were ≥ 95% reversible with equilibrium denaturation curves fitted to a two-state model, derived via modification of the Gibbs–Helmholtz equation [6], [45], [46], to yield the melting temperature (*T*_m_). Melting profiles for heterodimers are clearly distinct from averages of constituent homodimeric melts (shown in Table 1 and via dimer exchange in Fig. 3b), indicating that helices form heterodimeric complexes, with the cooperative nature of the melting profiles suggesting an apparent two-state process. *T*_m_ values were determined by least-squares fitting of the denaturation assuming a two-state folding model that is widely used for coiled coils [46] and provided an excellent fit to our data.

### Size-exclusion chromatography

Size-exclusion experiments were performed at room temperature using a Superdex Peptide 10/300 GL column (GE Healthcare Life Sciences) by injecting 100 μl of a 50 μM (total peptide concentration) sample in 10 mM potassium phosphate and 100 mM potassium fluoride, pH 7, at a flow rate of 0.5 ml/min. Elution profiles were recorded via *A*_280_.

### Mass spectrometry

All samples were analysed using ultrahigh-resolution time-of-flight MS (MAXIS, Bruker Daltonik GmbH) using a syringe pump at a flow rate of 3 μl/min and using 50 μM Pt samples. MS detection was in positive ion mode with a mass range of 100–2000 *m*/*z* and a spectral rate of 1.0 Hz.

The following are the supplementary data related to this article.

Supplementary material

Fig. S1 Helical wheel diagrams for all thirty six possible pairs within the selected eight-peptide interactome. Shown are (a) hypothetical pairs formed by peptide 1-4, (b) hypothetical pairs formed by peptide 5-8 and (c) hypothetical (undesirable) interactions formed between peptides 1-4 and 5-8. Electrostatic attractions and repulsions are shown via blue and red hashed lines, respectively. Diagrams were generated using DrawCoil 1.0, http://www.grigoryanlab.org/drawcoil.

Fig. S2 Scatter diagrams for predicted *versus* observed thermal melting values. Overall the correlation is poor (black line fitted to all 36 data points; *r*^2^ = 0.26). The correlation between peptides 5 and 8 is also poor (blue line fitted to 10 blue data points; *r*^2^ = 0.29); however, the correlation between peptides 1 and 4 is very good (red line fitted to 10 red data points; *r*^2^ = 0.70).
