# Supplementary material for: Deriving Heterospecific Self-Assembling Protein–Protein Interactions Using a Computational Interactome Screen
Source: J Mol Biol. 2016 Jan 29;428(2Part A):385–98. doi: 10.1016/j.jmb.2015.11.022 (PMC4751974; doi:10.1016/j.jmb.2015.11.022)
Supplement: Supplementary file 1 — Supplementary material [file mmc1.docx]

***Including Double Asn cores* –** Owing to the computational requirements of screening the large number of sequences interactions and pairs, it was decided to limit sequences in the libraries to only those sequences which contained two Asn residues and two Ile residues. This reduced the possible number of core combinations from 16 to 6, and consequently the number of peptides entering the interactome screen was reduced to 37.5% and therefore the interactome to 14.1% (i.e. from 4096 peptides to 1536, and the interactome size from 8,390,656 to 1,180,416). By limiting the core in this way, if Asn and Ile residues were aligned within the interactions but each pair of interactions contained different Asn combinations this would favour interaction specificity by ensuring that at least two of the core Asn/Ile residues were misaligned in any undesired interactions. As well as saving computation time, inclusion of two Asn residues was deemed to be likely to provide considerable specificity benefits (*1*). Sequences which contain only one of the four core positions occupied by Asn residues may have at most two Asn-Ile misalignments. By including a second Asn residue, it is possible to have Asn-Ile misalignments at all core residues, by inversion of the arrangement of Ile and Asn residues in the core positions between non-interacting pairs. Even though inversion can only produce such a high degree of specificity against one set alternative core combination, there is also the option for a partial inversion of part of the core sequences, which would cause a misalignment of 50% of the core residues. In summary, the inclusion of two Asn and two Ile residues in library sequences can equal and exceed the specificity offered by inclusion of a single Asn residue, and also allow the algorithm to search for sequences within a more viable search space. This together with Glu-Lys options at each ***e*** and ***g*** position led to a complete library size of 1536 members.

***Software -*** The following tools are listed in the order that have been used to derive the heterospecific pairs listed in the manuscript.

1. [***Generate Library Sequences***](http://people.bath.ac.uk/jm2219/biology/create-library.htm) (<http://people.bath.ac.uk/jm2219/biology/create-library.htm>) generates a list of all given sequences within a peptide library. Data can be entered at the peptide (where a ‘?’ defines each position to be scrambled with specific options for each ‘?’ entered by the user) or DNA level (where libraries are built using degenerate codons (e.g. the codon WSG = options of S,T,W,R).
2. [***bCIPA Interactome Screen***](http://people.bath.ac.uk/jm2219/biology/bcipa-interactome.htm) (<http://people.bath.ac.uk/jm2219/biology/bcipa-interactome.htm>) screens all interactions between a defined set of sequences (generated from i) and outputs a predicted Tm value for each interaction. This includes an option to provide a colour coded heat-map of the resulting interactome. In the case of our 1536 member library this represented 1,180,416 hypothetical PPIs within the interactome.
3. [***Find Pairs / Find Quadruples***](http://people.bath.ac.uk/jm2219/biology/find-pairs.php) (<http://people.bath.ac.uk/jm2219/biology/find-pairs.php>) works using the [***bCIPA Interactome Screen***](http://people.bath.ac.uk/jm2219/biology/bcipa-interactome.htm) engine. It allows users to input a library of sequences and then screen the resulting interactome to identify two (i.e. four peptides - pairs), four (i.e eight peptides - quadruples), or even higher numbers of sets of leucine zippers that are specific within each other’s presence. Users can also input desired parameters for their specific pairs (maximum homodimer T_m_, minimum desired T_m_, maximum undesired T_m_, minimum delta T_m_) depending on their requirements. The software is incremental, meaning that options for ‘find quadruples’ will appear within the software once ‘find pairs’ has identified sequences that meet the given requirements. Throughout, the user is able to control stringency by inputting the necessary parameters. These include the desired ΔT_m_, maximum undesired T_m_ and maximum desired T_m_. The output will then list all T_m_ values within the resultant eight-peptide interactome and generate a heatmap (Figure 2). Required parameters for screening the complete 1536 peptide interactome screen are listed in the methods section and are designed to keep homodimeric and off-target T_m_ values low, and desired T_m_ values high. Having screened all 1,180,416 hypothetical interactions we were able to identify pairs, and later quadruples of coiled coils that met the specificity criteria. Once sequences were identified within ***Find pairs***, the ***Find quadruples*** options were available to identify sets of pairs that retained specificity when combined into quadruples. Again, the user is able to input a maximum tolerated off-target T_m_ value as well as a minimum difference in T_m_ between desired and non-desired pairs.

**Figure S1:** Helical wheel diagrams for all thirty six possible pairs within the selected eight-peptide interactome. Shown are ***a)*** hypothetical pairs formed by peptides 1-4 ***b)*** hypothetical pairs formed by peptides 5-8 and ***c)*** hypothetical (undesirable) interactions formed between peptides 1-4 with 5-8. Electrostatic attractions and repulsions are shown via blue and red hashed lines respectively. Diagrams were generated using DrawCoil 1.0, <http://www.grigoryanlab.org/drawcoil>.

**Figure S2:** Scatter diagrams for predicted *vs.* observed thermal melting values. Overall the correlation is poor (black line fitted to all 36 data points; r^2^ = 0.26). The correlation between peptides 5-8 is also poor (blue line fitted to 10 blue data points; r^2^ = 0.29), however the correlation between peptides 1-4 is very good (red line fitted to 10 red data points; r^2^ = 0.70).

References

1. Lumb, K. J., and Kim, P. S. (1995) A buried polar interaction imparts structural uniqueness in a designed heterodimeric coiled coil, Biochemistry 34, 8642-8648.
