# Supplementary figures and images for: Deriving Heterospecific Self-Assembling Protein–Protein Interactions Using a Computational Interactome Screen
Source: J Mol Biol. 2016 Jan 29;428(2Part A):385–98. doi: 10.1016/j.jmb.2015.11.022 (PMC4751974; doi:10.1016/j.jmb.2015.11.022)

Figure S1a

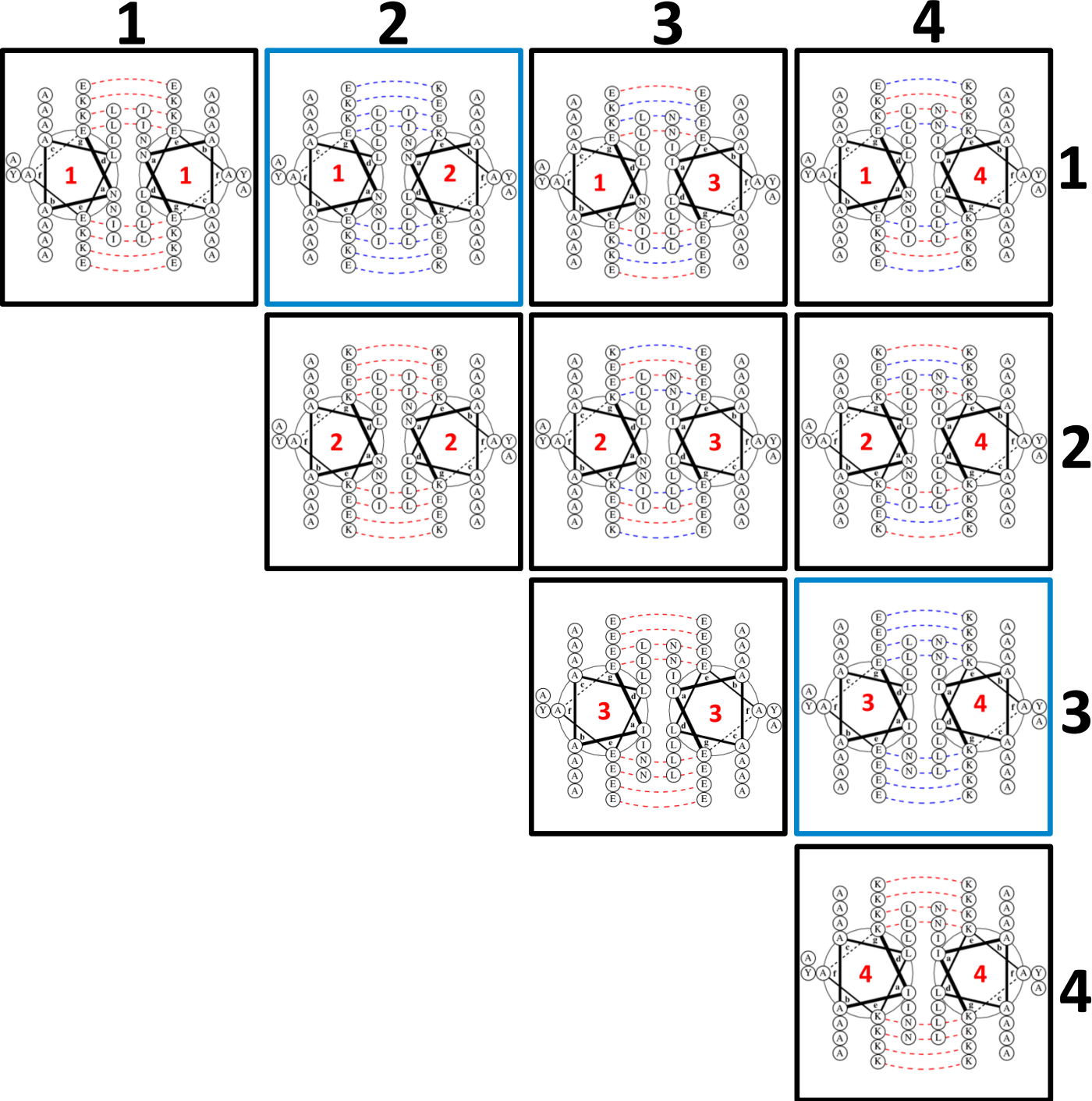

Figure S1b

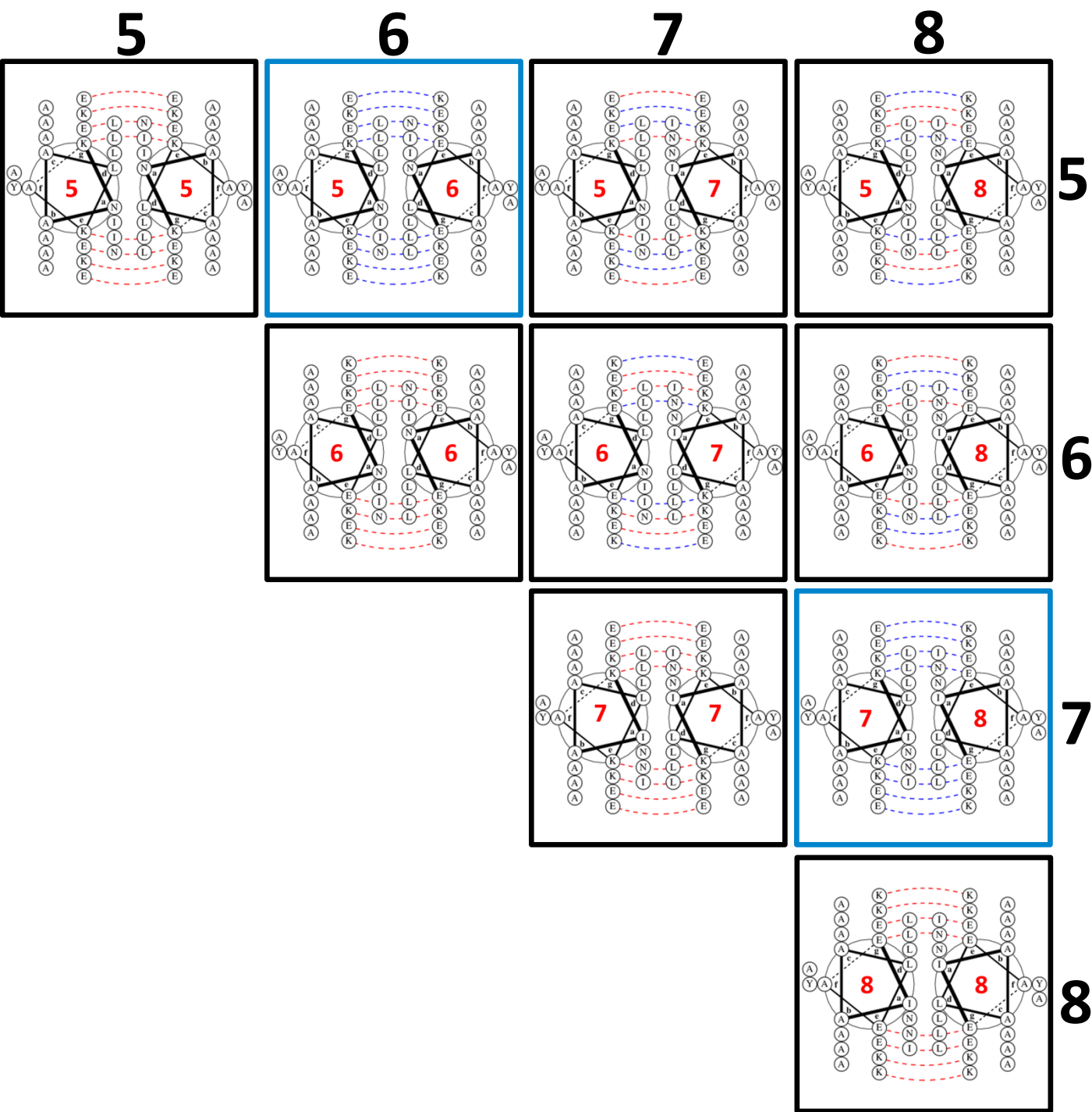

Figure S1c

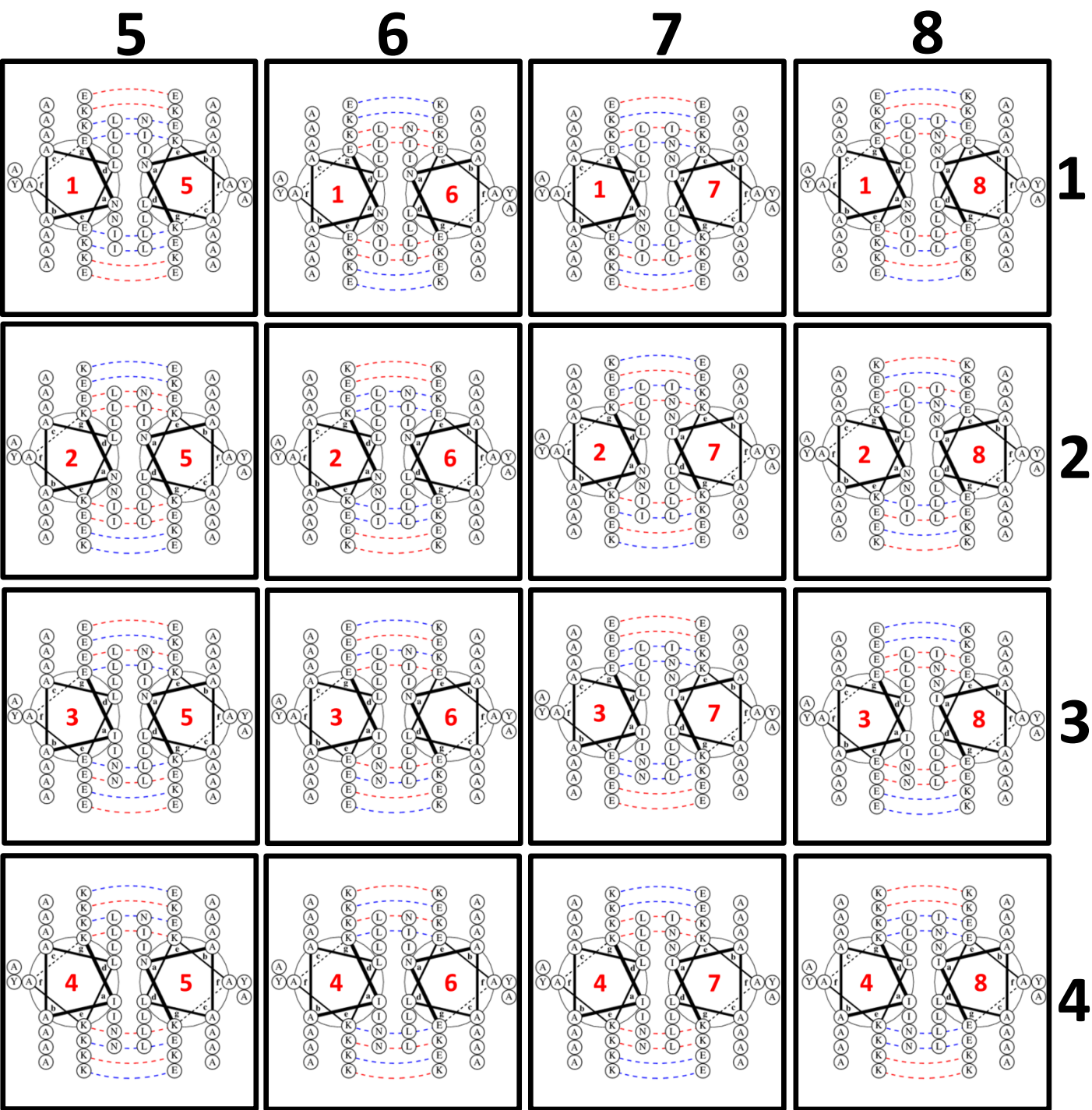

Supplement: Fig. S1 — Helical wheel diagrams for all thirty six possible pairs within the selected eight-peptide interactome. Shown are (a) hypothetical pairs formed by peptide 1-4, (b) hypothetical pairs formed by peptide 5-8 and (c) hypothetical (undesirable) interactions formed between peptides 1-4 and 5-8. Electrostatic attractions and repulsions are shown via blue and red hashed lines, respectively. Diagrams were generated using DrawCoil 1.0, http://www.grigoryanlab.org/drawcoil. [file mmc2.pdf]

Figure S2

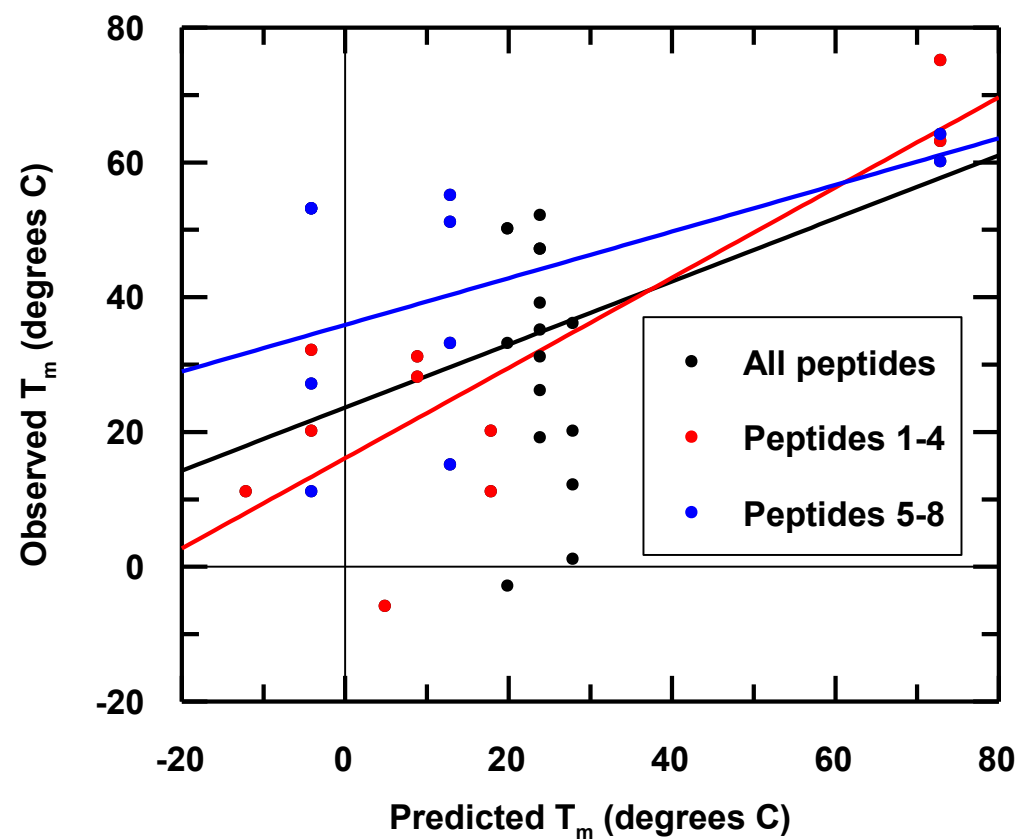

Supplement: Fig. S2 — Scatter diagrams for predicted versus observed thermal melting values. Overall the correlation is poor (black line fitted to all 36 data points; r2 = 0.26). The correlation between peptides 5 and 8 is also poor (blue line fitted to 10 blue data points; r2 = 0.29); however, the correlation between peptides 1 and 4 is very good (red line fitted to 10 red data points; r2 = 0.70). [file mmc3.pdf]
